# Cognitive Load Changes during Music Listening and its Implication in Earcon Design in Public Environments: An fNIRS Study

**DOI:** 10.3390/ijerph15102075

**Published:** 2018-09-21

**Authors:** Eunju Jeong, Hokyoung Ryu, Geonsang Jo, Jaehyeok Kim

**Affiliations:** 1 Department of Arts and Technology, Hanyang University, Seoul 04763, Korea; hryu@hanyang.ac.kr (H.R.); azureluna@hanyang.ac.kr (G.J.); 2 Division of Industrial Information Studies, Hanyang University, Seoul 04763, Korea; 3 Graduate School of Technology and Innovation Management, Hanyang University, Seoul 04763, Korea; 4 Department of Industrial Engineering, Hanyang University, Seoul 04763, Korea; jhyeok.kim@letinar.com

**Keywords:** melody perception, contour identification, auditory attention, cognitive load, near-infrared spectroscopy, earcon design

## Abstract

A key for earcon design in public environments is to incorporate an individual’s perceived level of cognitive load for better communication. This study aimed to examine the cognitive load changes required to perform a melodic contour identification task (CIT). While healthy college students (*N* = 16) were presented with five CITs, behavioral (reaction time and accuracy) and cerebral hemodynamic responses were measured using functional near-infrared spectroscopy. Our behavioral findings showed a gradual increase in cognitive load from CIT1 to CIT3 followed by an abrupt increase between CIT4 (i.e., listening to two concurrent melodic contours in an alternating manner and identifying the direction of the target contour, *p* < 0.001) and CIT5 (i.e., listening to two concurrent melodic contours in a divided manner and identifying the directions of both contours, *p* < 0.001). Cerebral hemodynamic responses showed a congruent trend with behavioral findings. Specific to the frontopolar area (Brodmann’s area 10), oxygenated hemoglobin increased significantly between CIT4 and CIT5 (*p* < 0.05) while the level of deoxygenated hemoglobin decreased. Altogether, the findings indicate that the cognitive threshold for young adults (CIT5) and appropriate tuning of the relationship between timbre and pitch contour can lower the perceived cognitive load and, thus, can be an effective design strategy for earcon in a public environment.

## 1. Introduction

Earcon has been defined as “abstract, synthetic tones that can be used in structured combinations to create auditory messages” [1,2] and can represent almost any type of event or interaction in public environments [3]. Earcon takes advantage of the auditory modality, which is omnidirectionally perceivable and reaches across distances, thus making it preferable to other sensory modalities, for example, in situations where visual information is unavailable or not fully available [4]. When individuals are faced with a high visual cognitive load or the inaccessibility of visual information, auditory earcons can deliver information [5,6,7], thus facilitating almost immediate decision-making and action [8]. An assumed disadvantage of non-verbal and non-iconic auditory earcon is that their meaning might be vague and require more time to learn [9].

In a real-world auditory context, multi-layered sound streams are concomitant (e.g., voices, natural sounds, and electronic noises). Especially in public environments, where high levels of background speech or noises are present, communication challenges are common [10,11,12]. Earcon can be the best option for successful communication and decision-making [13,14], and be preferable even over speech-based auditory display [15,16], which requires a longer duration to distinguish the source from environmental sounds or to completely understand the sentence [17,18].

It is a natural phenomenon that we attend to certain features of sounds and segregate meaningful information from complex auditory scenes [19,20]. In this process, we are compelled to use a combined strategy of attention, which embraces a broad range of subtypes, such as stimulus orientation, vigilance, selective attention, and executive control [21,22,23,24,25]. As the complexity of auditory environments increases, individuals tend to utilize more attentional resources to effectively process task-relevant against task-irrelevant information (e.g., the cocktail party effect [26,27,28]), resulting in increased mental effort. In this sense, the cognitive load theory is applicable to auditory attention in public environments [19,20].

The load theory of attention [29,30,31,32] proposes that the level and type of information provided in a task can determine the degree of mental effort. More specifically, stimulus complexity (i.e., perceptual demand) and task difficulty (i.e., cognitive control load) are believed to generate and impose different amounts of cognitive load [30,31,32,33]. Perceptual demands are perceived cognitive loads caused by the stimulus complexity, which further emphasizes the “bottom-up” aspects of information processing, while cognitive control demands are associated with the “top-down” aspects of cognitive control, such as cognitive flexibility, working memory, and executive functioning [33,34,35].

Many studies have suggested that cognitive load is the defining factor for designing an efficient and effective earcon [36,37,38]. The existing approach to earcon design, specific to multiple and concurrent earcons in auditory display, focuses on the issues around masking and interfering, providing solutions that utilize spatially disparate sound locations [39,40,41]. It is the assumption that the allocation of information to the auditory field of view can increase the efficiency in attentional resource management and thus contribute to reducing cognitive loads [42,43,44]. Without spatial separation, the multiple sound sources will have a greater tendency to fuse together, making them more difficult to understand [45].

Various acoustic features are also of great importance in controlling cognitive loads [16,46]. The core features include amplitude (loudness), frequency (pitch), and waveform (timbre). Loudness is a dominant characteristic of auditory perception and provides a strong affordance to assign a considerable amount of attentional resource [47]. An auditory alarm system, for example, recommends a sound level of at least 75 dB and allows decibel increases in a very emergent situation. However, sounds that are more than 140 dB or in the case of a sudden increase of more than 30 dB can cause emotional displeasure and sudden fright [48,49]. Loudness control seems less effective and informative, so applying other perceptual features such as pitch and timbre have been recognized as potentially useful.

Using a combination of timbre and pitch is more promising in earcon design. Recently, researchers have reported the interaction effects between two perceptual features. In particular, pitch and timbre showed a symmetric relationship, so modulation in the spectral feature of timbres yields changes in pitch recognition [50]. Li et al. [51] reported enhancement in pitch identification when provided with timbre information. When more perceptually distinguishable timbres were used, there was a significant improvement in performance of the identification task [52]. Collectively, the findings suggested that auditory events and their constituent acoustic features (i.e., bottom-up process) can influence cognitive load changes by increasing the chance to establish prediction or anticipation [53]; thus, designing the features are of importance.

In fact, earcons are presented in a simple music-like pattern [3] and are experienced as melody. Given that concurrent earcon patterns are very similar to music where multi-layered streams (e.g., two melodies, one melody and accompaniment) convey aesthetic and meaningful information, we can integrate neurocognitive evidence indicating the attention system in the brain involving melody perception. Previous studies have revealed that melody perception leads to voluntary and involuntary activation of the brain [54,55,56,57,58,59]. Especially in multi-voice music-listening, in which diverse musical streams are presented concurrently, neural activation seems to be differentiated by the type of musical texture and the manner of listening (e.g., holistic, selective, or divided) [47,48,49,50]. A group of studies employing various types of musical tasks (e.g., error detection and target tone identification) have also reported common but specific neural activation depending on the given stimulus and task [60,61,62]. Such findings indicated that listening to music in various contexts (e.g., polyphonic music) can utilize and activate multiple attention systems in the brain, which are associated with the level of cognitive load. Although the existing literature on music and neuroscience indicates that various features may provide a hint toward the reduction of cognitive load (i.e., automatic or involuntary processing), how much cognitive load the other features of sound, such as pitch contour and timbre or their combination, would impose is still open to question.

Several studies have employed earcons presented simultaneously and examined their effect on cognitive loads. Concurrent spatialized sounds, for example, were used in a mobile-based system in Nomadic Radio [63]. Gaver et al. [64,65] used concurrent presentation of auditory information to determine the status and monitor the vital processes of plants. More recently, McGookin et al. [2] emphasized the importance of stimulus and task characteristics in designing earcon. The authors examined the effect of the characteristics on task performance and found that the amount of auditory information presented within the same time window and the acoustic properties (e.g., timbre) can together influence overall task performance. Although the effectiveness of concurrent earcon has been evaluated by assessing behavioral performance, the physiological aspects of cognitive load and its relationship with behavioral aspects has rarely been examined so far.

In the present study, we focused on cognitive loads associated with the subtypes of attention during a melodic contour identification task (CIT). In our previous studies [66,67], we developed a method of music-based attention assessment to examine the attentional function of individuals with a moderate-to-severe level of traumatic brain injury (TBI). Findings from our first study indicated that melodic contour identification was feasible both for healthy adults and adults with TBI, and had a high reliability (split-half coefficient = 0.836, Cronbach’s *α* = 0.940) [67]. In the second study, Jeong [66] investigated construct validity using factor analysis, yielding four factors of attention. The overall findings imply that the melodic CIT can distinguish different types and levels of auditory attention and, thus, is a valid and reliable task.

Here, we measured hemodynamic changes in the frontopolar area (Brodmann’s area 10 (BA10)) to examine cognitive loads associated with CITs. This area receives information from the primary auditory cortex and relays the same to other regions of the prefrontal cortex (PFC). Laguë-Beauvais et al. [68] reported that this area receives both verbal and non-verbal information from the superior temporal gyrus. This area generally modulates attention-related function and becomes more active as the task complexity increases. Plakke and Romanski [69] indicated that BA10 manages audition-related task performances that require heavy cognitive processing. Previous studies have not measured the cognitive load imposed during different types of auditory attention, which would be important in public earcon design. For this purpose, the present study used functional near-infrared spectroscopy (fNIRS), a recent advancement in brain imaging technology, to measure cognitive load changes in the prefrontal regions [68,70,71,72].

Designing a nonverbal auditory earcon using both pitch and timbre requires the understanding of how such musical components impose cognitive loads. The present study, thus, aimed to determine the cognitive loads required to perform CITs, using pitch contours in conjunction with timbre in a simulated real-world auditory environment. With a multi-faceted approach to cognitive load, we assessed behavioral data along with hemodynamic changes using fNIRS. The neurophysiological response complements the sensitivity of the behavioral data and is a good indicator for decision-making in relation to perceived sensory information [35,73].

## 2. Materials and Methods

### 2.1. Participants

Sixteen college student volunteers (10 men and 6 women), who were not majoring in music, were recruited from a university in Seoul, Republic of Korea. We intentionally controlled the level of the participants’ general education and musical experience. All volunteers were freshmen or sophomores, limiting the years of education to 13 or 14 years. None of the participants were professionally trained in music (less than 1 year of professional music training), nor did they have a neurological medical history or any sensory impairment. The mean age of the participants was 23.5 years (standard deviation = 1.7 years).

### 2.2. Music Stimuli

**Figure 1:**
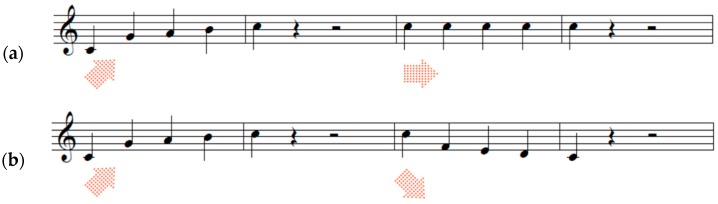
Samples of melodic contours (adopted from [66,67]). Item (**a**) includes ascending and stationary contours and Item (**b**) includes ascending and descending contours.

As shown in Figure 1, melodic contours are a series of tones moving in different directions (i.e., ascending, descending, and stationary). Two different types of contour were combined consecutively to yield six types of test items (i.e., ascending and descending, ascending and stationary, stationary and ascending, stationary and descending, descending and ascending, descending and stationary). The presentation time of each test item was 5250 ms, including two contours (2250 ms for each) and inter-contour interval (750 ms). Figure 1 shows examples of the pitch contours used in the study.

**Figure 2:**
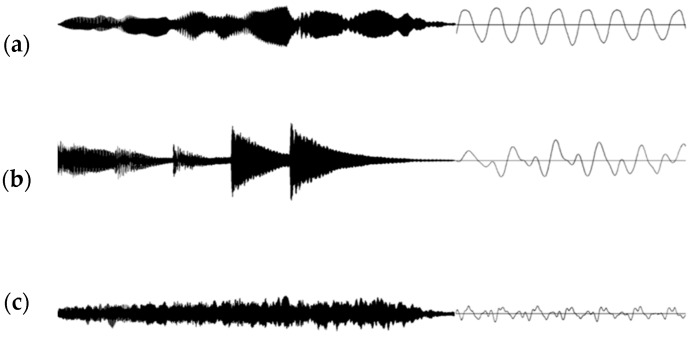
Waveforms of instrument timbres. The instruments are (**a**) flute, (**b**) piano, and (**c**) strings.

**Figure 3:**
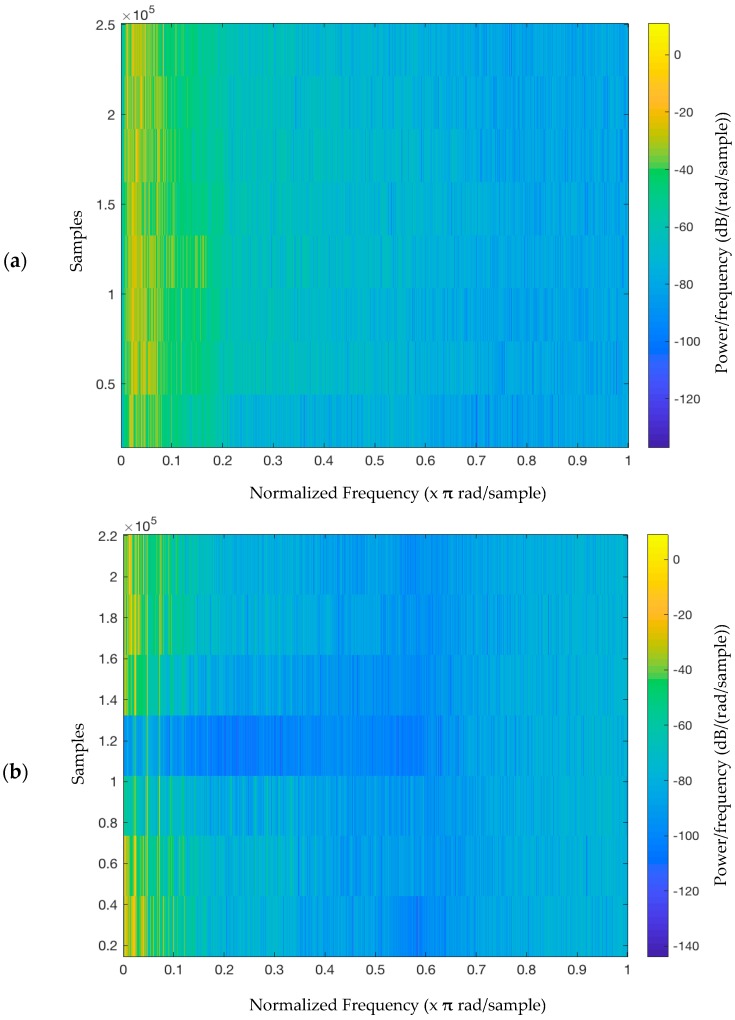
Spectrogram of target contours presented with (**b**) environmental noise and (**b**) a target-like distractor (**b**).

The timbres of the three instruments have different spectral and temporal complexities. The flute has a relatively simple spectrum with little attack; the piano has a more complex spectrum with a sharp attack; the strings have a complex spectrum with a soft attack (Figure 2). We selected the instruments following a previous study that classified various musical instruments based on the spectral features of timbre, such as the harmonic structure, inharmonicity, and harmonic energy skewness [74]. Figure 3 shows a spectrogram of target contours presented combined with environmental noise (Figure 3a), and with a target-like distractor (Figure 3b) using a short-time Fourier transform. Signal-to-noise ratio (SNR) was 7.2385 for the target contour presented by the flute against environmental noise and 8.0835 dB for the target contours with target-like contour, for example.

The six types of test items were modulated in five different keys (G# to C major) and presented with three instrument timbres, yielding a total of 90 test items. In each CIT, participants were randomly presented with 18 out of 90 items and asked to identify the directions of contours. The melodic contours were generated by a musical instrument digital interface (MIDI) synthesizer (YAMAHA DGX 230, Hamamatsu, Japan) with a digital audio workstation (Logic Pro X, Apple Inc., Cupertino, CA, USA). The experimental test was developed as a computerized version, using Visual Studio (Microsoft, Washington, DC, USA).

### 2.3. Contour Identification Task

The computerized version of the CIT was designed to measure different types of auditory attention and the associated cognitive load changes (Table 1). The task stimuli and structures were adopted from previous studies [66,67,75] and modified for the current purpose. In CIT1, two consecutive contour directions were presented as a target contour without distraction (i.e., focused identification). Ten types of environmental sounds, including traffic, raining, twittering, ticktack, bustling, laughing, gabbling, applause, crying, and jeering sounds were randomly presented against target contours in the second task (i.e., CIT2, selective identification against environmental noise). Both CIT3 and CIT4 simultaneously presented two melodic contour streams. For CIT3, the participants were asked to attend to a target musical stimulus while ignoring the target-like distraction (i.e., selective identification against a more competing distractor). Target-like distractors had the same or different direction of contour and were played using different musical instruments. The CIT4 was more complex since the task involved an alternating focus between two melodic contours. Participants were asked to intentionally shift and re-focus their attention between the two auditory stimuli. Lastly, in CIT5, participants were asked to divide their attentional focus over both melodic contours and to completely identify all four contour directions.

### 2.4. fNIRS Data Acquisition and Pre-Processing

**Figure 4:**
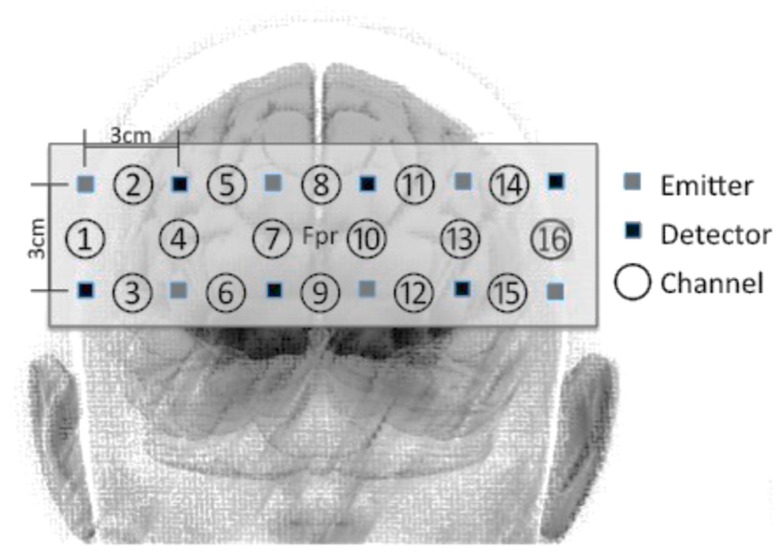
Placement of emitters, detectors, and channels for Spectratech OEG-16. Fpr refers to the frontopolar region.

In this study, we used fNIRS (16-channel Spectratech OEG-16, Yokohama, Japan) to measure hemodynamic changes, which is assumed to indicate the cognitive loads involved in each CIT [76,77,78] The center of the measurement unit was placed between the frontopolar areas (Fp1 and Fp2), according to the international 10–20 system (Figure 4). The task-related hemodynamic changes were recorded through 16 channels, with a sampling rate of 0.65 s. The hemodynamic changes that were measured included oxygenated hemoglobin (HbO_2_), deoxygenated hemoglobin (HHb), and the sum of oxygenated and deoxygenated hemoglobin (HbT).

The fNIRS data were collected and converted into concentration changes of hemoglobin using the modified Beer-Lambert law. In general, raw fNIRS data are affected by other physiological signals, such as the heart rate, breathing, and eye-blinking; therefore, a zero-phase low- and high-pass filter with cut-off frequencies of 0.01–0.09 Hz was applied, using MATLAB (The Mathworks Korea LLC, Gangnam-gu, Korea), to pre-process the raw NIRS signals [79,80,81]. The values were standardized by subtracting the mean values of HbO_2_, HHb, and HbT obtained during the first 20 s baseline period, which is obtained prior to any type of stimulus presentation, from the means of each of the five CITs. This was performed since each individual had different baseline values of oxygen metabolism [81].

### 2.5. Procedure

The study was conducted in accordance with the Declaration of Helsinki, and the protocol was approved by the Institutional Review Board of Hanyang University (HYI-141273). The experiment was announced to college students registering for the course, “An Introduction to Cognitive Psychology”. All recruitment processes were performed electronically, and 16 out of 20 volunteers participated in this study. Three were excluded because of their professional experience in music training. One left-handed volunteer was also excluded. Informed consent was obtained from all individual participants included in the study. Once participants agreed to participate voluntarily and provided full permission for the publication, they filled out a nine-item demographic questionnaire (age, sex, academic major, previous musical experience, etc.).

A band-type NIRS containing an array of 12 probes (i.e., emitters and detectors) was attached to each participant’s forehead. The probes were connected to the main board of the NIRS, which was connected to a computer. Auditory stimuli were delivered diotically via headphones at a constant volume, and visual cues specifying the target musical stimulus were presented on a monitor. A 20 s baseline was recorded prior to stimulus presentation, before and after each of the five CITs, while the participants fixed their eyes on the center of the monitor.

The experiment started with an initial familiarization session. All participants took part in a brief stimulus-familiarization phase. In a practice session, participants were asked to identify the directions of the contours until they could correctly identify more than 80% of the directions. In a main session, each of the five CITs started with a brief instruction in terms of the task characteristics given in each CIT and how to respond to test items. Participants were also instructed to identify the directions of the target contours by clicking the arrow corresponding to the contour direction, as accurately and immediately as possible.

**Figure 5:**
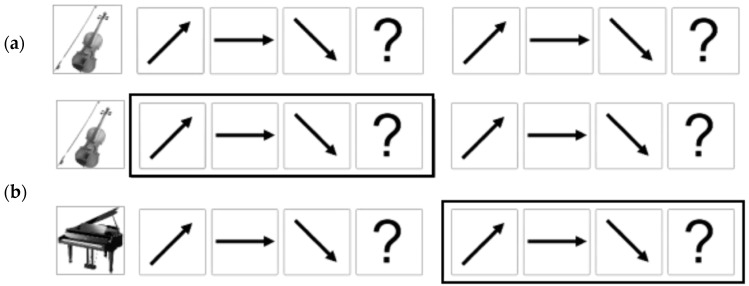
Examples of answer pages. (**a**) is given in CIT3 and (**b**) is given in CIT4. The boxes were presented prior to stimulus presentation (CIT4), while they appeared after the stimulus presentation (CIT5).

In terms of a visual cue, CIT1 and CIT2 had no visual cues; however, in CIT3, a picture of an instrument that plays target contours was presented prior to presenting the item to indicate which contour the participants selectively listened to. In CIT4, outlined boxes were additionally used to hint at which contours the participants selectively listened to and shifted from one to another instrument (see Figure 5). For example, the first outlined box appeared in the upper or lower line with the first set of contours, and the second box appeared with the second set of contours. In CIT5, the two boxes appeared in a random manner after the test item presentation, so the participants were asked to attend and hold information from four contour directions, but identified two of them (i.e., the contours of which the outlined boxes appeared).

The participants were also informed that their behavioral and hemodynamic responses were being recorded throughout the experiment. Reaction time was the sum of overall times between post-stimulus to the first arrow selection and between the first arrow selection and the second arrow selection. In each CIT, a total of 18 test items were presented (a blocked design) and the order of CIT was randomized across participants. When participants identified directions of all contours correctly, one point was assigned, so participants could obtain a maximum of 18 points for each CIT. The main experimental session required approximately 30 min to complete and was performed in a sound-proof room to control for other noises. The ambient light and temperature remained constant throughout the experimental sessions.

### 2.6. Statistical Analysis

We used a repeated measures design for the statistical analysis. The independent variables were the CITs (CIT1, CIT2, CIT3, CIT4, and CIT5) for the behavioral analysis and the sessions (pre-baseline, CIT1, CIT2, CIT3, CIT4, CIT5, and post-baseline) for the fNIRS analysis. The dependent variables were behavioral responses (performance accuracy and reaction time) and hemodynamic responses (HbO_2_ and HHb). For behavioral analysis, the rate corrected score (RCS) was estimated using c/ΣRT, in which c refers to correct responses and RT refers to response time [82,83]. We selected the following NIRS channels representing the frontopolar area (BA10, BA11), including Channels 7, 8, 9, and 10. All statistical analyses were performed using the repeated measures analysis of variance (ANOVA) in SPSS version 20 (SPSS Inc., Chicago, IL, USA).

## 3. Results

### 3.1. Accuracy and Response Time

The behavioral responses for the five CITs are shown in Table 2. The mean accuracy was almost perfect for CIT1 (97%), followed by CIT2, with environmental noise (96%); CIT3, with a target-like melodic distractor (92%); and CIT4, with two alternating targets (89%). As expected, accuracy was the lowest (67%) for the divided identification of two concurrent target contours (CIT5). A similar trend was found for reaction times across the CITs. A gradual increase was observed until CIT3 (ranging from 2896 to 2999 ms), followed by a large increase at CIT4 (3907 ms). The time required to complete CIT5 was the longest (7825 ms). 

For further analysis, we performed a non-parametric Friedman test since performance accuracy and reaction time showed non-normality in distribution (Shapiro–Wilk test, *p* < 0.05). Results of accuracy rendered a chi-square value of 52.80 (*p* < 0.001). Nemenyi post-hoc analyses showed a significant difference between CIT1 and CIT4 (*p* < 0.05), CIT1 and CIT5 (*p* < 0.001), CIT2 and CIT5 (*p* < 0.001), CIT3 and CIT5 (*p* < 0.05), and CIT4 and CIT5 (*p* < 0.05). Results of reaction time rendered a chi-square value of 47.25 (*p* < 0.001). Nemenyi post-hoc analyses showed a significant difference between CIT1 and CIT5 (*p* < 0.001), CIT2 and CIT4 (*p* < 0.01), CIT2 and CIT5 (*p* < 0.001), and CIT3 and CIT5 (*p* < 0.001).

**Figure 6:**
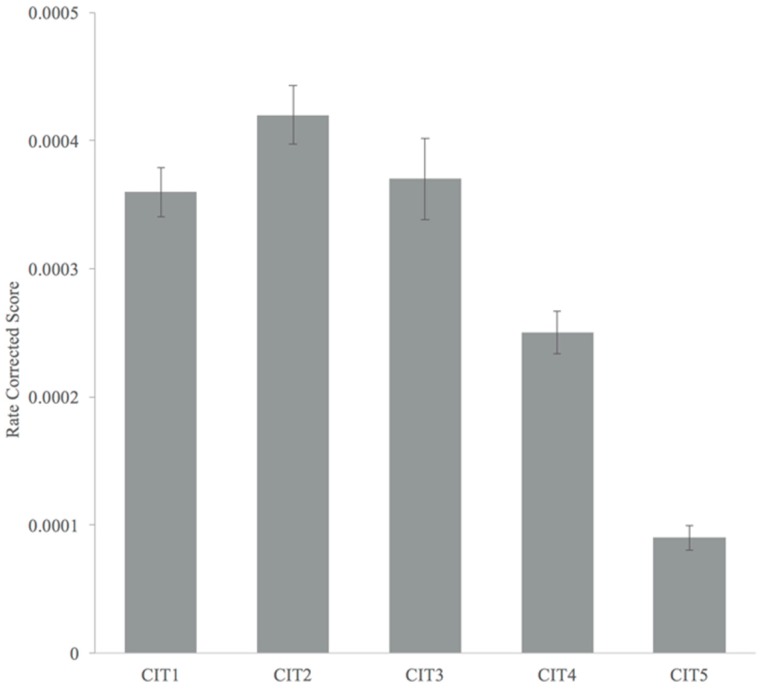
Changes in rate corrected scores across contour identification tasks (CITs).

In order to clarify the trend in behavioral responses, RCSs were calculated. We confirmed normality of the data using Shapiro–Wilk test (*p* > 0.05). A repeated measures ANOVA showed a significant main effect of CIT on the RCS (*F*_(4,60)_ = 61.189, *p* < 0.001), indicating that performance worsened as the CIT became more difficult. Pairwise post-hoc analyses with Bonferroni correction revealed that the differences between CIT1 and CIT2, CIT2 and CIT3, and CIT1 and CIT3 were not statistically significant (*p* > 0.05). All remaining pairwise tests revealed significant differences, including those between CIT3 and CIT4 (*p* < 0.001) and between CIT4 and CIT5 (*p* < 0.001). These findings indicated that the five CITs could be organized into three groups. Briefly, CIT1, CIT2, and CIT3 tended to be grouped together, while CIT4 and CIT5 showed clear distinctions in terms of difficulty (Figure 6).

Altogether, our behavioral findings showed a clear distinction among the CITs. Accuracy decreased and reaction time increased as the CITs became more complicated (CIT4 and CIT5). More importantly, the behavioral responses implied that CIT4 is sensitive to the cognitive threshold of healthy young adults. Furthermore, CIT5 is the most challenging task in terms of general attentional capability.

### 3.2. Hemodynamic Responses

**Figure 7:**
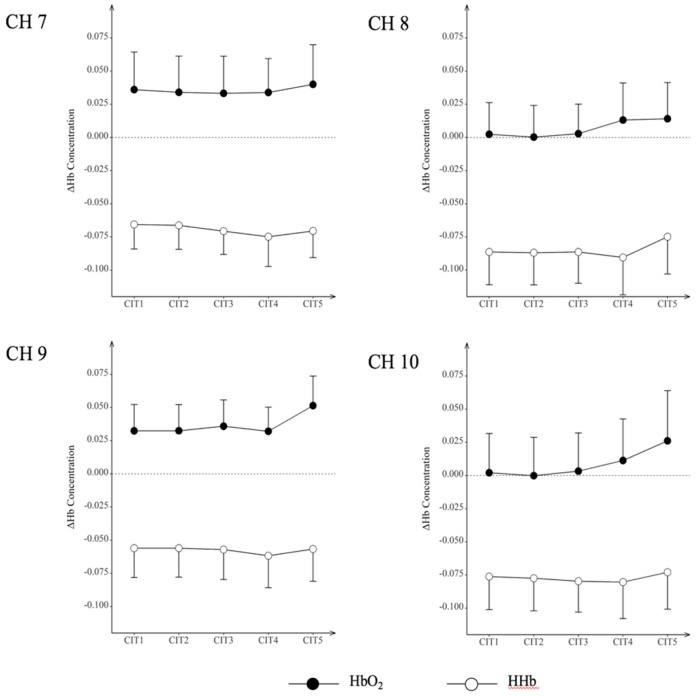
Changes in HbO_2_ and HHb across contour identification tasks. HbO_2_: oxygenated hemoglobin; HHb: deoxygenated hemoglobin.

We analyzed hemodynamic responses using HbO_2_ and HHb across the CITs obtained from the frontopolar areas (BA10 and BA11). Table 3 and Figure 7 presents the overall hemodynamic changes across these channels, i.e., HbO_2_ and HHb at Channel 7 (CH7), CH8, CH9, and CH10. Overall, HbO_2_ increased and HHb decreased while the level of CITs increased. Subsequently, we performed a repeated measures ANOVA to identify the channels that may indicate cognitive loads across CITs. The findings showed a significant main effect of CIT on HbO_2_ (*F*_(4,60)_ = 3.204, *p* < 0.05) in CH9. Post-hoc pairwise comparisons revealed that HbO_2_ increased significantly between CIT1 and CIT5, CIT2 and CIT5, and CIT4 and CIT5 (*p* < 0.05, respectively).

## 4. Discussion

The main purpose of this study was to examine how various components and textures of music can impose different levels of cognitive loads, as evidenced by behavioral and hemodynamic responses. In our behavioral findings, accuracy and reaction times together indicated that the CITs can impose different levels of cognitive load. A gradual increase in RCS from CIT1 to CIT3 was followed by an abrupt increase between CIT3 and CIT4, and again between CIT4 and CIT5. The hemodynamic findings showed a significant increase between CIT1 and CIT5, CIT2 and CIT5, and CIT4 and CIT5. Collectively, the findings indicate that the perceived cognitive load can differ depending on the type of properties of the auditory stimuli and the manner of organizing the stimuli. Individuals can process two simultaneous auditory information provided in a selective attention task (i.e., CIT3) without an increase in cognitive load, while significant increases in cognitive load were observed in processing two auditory stimuli provided in a shifting or divided attention task (i.e., CIT4 and CIT5). At CH9, hemodynamic changes between CIT3 and CIT4 were non-significant; however, those at CH7, CH8, and C10 showed an overall increasing tendency, suggesting overall increases in cognitive load in the prefrontal regions.

### 4.1. Lessons from Behavioral and Hemodynamic Findings

Our findings revealed that healthy young adults performed tasks involving single-target melody perception (CIT1 and CIT2) almost perfectly. Additional cognitive loads between CIT1 and CIT2 were not indicated despite environmental noise presented against target contours (note that accuracy was similar and that reaction time even showed non-significant decreases in CIT2). A similar tendency was observed in CIT3. The findings indicate that melodic contours without a distractor or with a non-target-like distractor can be autonomously processed by healthy young adults. These results are consistent with the lack of effort required to process melodic components, as evidenced in a mismatch negativity study and its magnetic counterpart [62,84,85], as well as in an event-related potentials study [57]. Moreover, melody perception against melody-like distractors (CIT3) did not increase cognitive loads, indicating that intact selective attention (inhibitory attention control over irrelevant stimuli) is characteristic of healthy young adults [86,87,88].

In more challenging tasks, such as CIT4, in which participants were asked to shift their attentional focus from one timbre to another (note that CIT3 did not require such a switch), performance deteriorated significantly, as evidenced by both reaction time and accuracy. The present findings are similar to those reported by previous studies, which demonstrated that shifting attention to a particular location or timbre increases cognitive load and perceived difficulty of the task [89]. When participants were presented with CIT5, in which melodic contour directions must be identified simultaneously, their task performance became much worse; CIT5 appeared to require participants to listen to the directions of melodic contours either in the manner of rapidly alternating or continuously applied attentional focus between multiple streams of CIT5 [60,90,91]. The ability to perform this has been reported to be the most difficult to achieve in the auditory modality, and both typical and clinical populations showed the worst performance in a similar type of auditory attention task [2,45,67,92,93].

In hemodynamic analysis, the current findings showed a significant increase in HbO_2_ and a non-significant but continuous decrease in HHb across CITs. Increases in HbO_2_ were prominent between CIT1 and CIT5, CIT2 and CIT5, and CIT4 and CIT5, indicating increases in cognitive load during CIT5 (i.e., divided attention task for CIT5). Decreases in HHb were accompanied with cognitive load changes associated with HbO_2_. In general, both indicators (i.e., increased HbO_2_ and decreased HHb) accompany each other, indicating an increase in cognitive load [94,95]. The increase in hemodynamic responses were obvious in the frontopolar area (BA10). This area is known for its role in modulating attention performance in the auditory modality. Laguë-Beauvais et al. [68] reported that the BA10 receives both verbal and non-verbal information from the superior temporal gyrus and becomes more active as the task complexity increases. Plakke and Romanski [69] also indicated that the BA10 manages auditory tasks that require heavy cognitive processing. Significant increases in HbO_2_ in the frontopolar areas, thus, indicated an increased involvement of these areas in performing the given auditory task with increased cognitive loads.

The current findings can be interpreted as follows: (1) together, the characteristics of auditory stimuli and tasks can generate different levels of cognitive loads, and (2) cognitive load changes in the auditory modality can be measured by the hemodynamic activation in the frontopolar areas. Further, they indicate that individuals (typically younger adults) easily perceive multiple melodic contours that are presented simultaneously and process them in a goal-directed manner (i.e., CIT3) without any perceived increase in the cognitive load, both behaviorally and hemodynamically. The performance was better than we expected; however, there certainly was a limited capacity of allocating cognitive resources involving auditory information in our real-world auditory surroundings, in which multi-layered sounds exist. Behavioral and hemodynamic responses to CIT5 are considered the potential markers of the highest cognitive capability in healthy young adults. Such cognitive threshold can further provide a guideline for earcon design in a public environment.

### 4.2. Lessons from Additional Analyses on Directional Congruence and Timbre Similarity

What drew our attention here is the cognitive load involved in the performance of CIT3 and CIT4, in which two melodic contours are presented simultaneously. As mentioned earlier, our auditory environments embrace multi-layered sound streams. This is not exceptional in public environments, in which various types of products and services are in operation. For example, during sound design in the context of human–robot interaction, there are multi-layered sound streams, including sounds of machinery platforms, monitoring, alarming, and feedback sounds [96]. Since each sound aims to deliver certain information, it is very important to consider the priority of the information, the individuals’ cognitive ability, and contexts. Our findings clearly indicate that CIT5 is not comparable to the other CITs. Hence, for earcon design for a public use, CIT3 and CIT4 were further examined to identify the features of evacuation earcon design that might trigger a perception advantage.

In CIT3 and CIT4, the target and distractor have either congruent or incongruent pitch contour direction. That is, two pitch contours moving in the same direction are considered congruent, and those moving in different directions are called incongruent. In a similar vein, pitch contours with similar timbres are considered similar. The timbre of the string and flute are similar in terms of temporal and spectral dimensions, whereas the piano is dissimilar to both the flute and string [85,86,87]. We, thus, performed an additional analysis to examine stimulus- and task-specific effects on behavioral performance. The items of CIT3 and CIT4 were recorded in relation to direction congruence and timbre similarity between target and target-like contours (Table 4).

A two-way ANOVA revealed a significant effect of direction congruence (*F*_(1,611)_ = 7.79, *p* < 0.01) but not a statistically significant difference based on timbre similarity (*F*_(1,611)_ = 1.71, *p* > 0.05). Interestingly, there was a significant interaction between direction congruence and timbre similarity (*F*_(1,611)_ = 4.93, *p* < 0.05), which suggests that direction identification of incongruent contours is affected by timbre similarity. When the directions between the target and the distractor were congruent, accuracy was maintained (mean, 0.97 vs. 1.00) irrespective of timbre similarity. However, when their directions were incongruent, accuracy was much lower when the timbre of the target and the distractor was similar (mean, 0.84 vs. 0.95). Taken together, the results indicate that both the pitch contour and timbre can be supportively used for earcon design.

### 4.3. Implications for Auditory Earcon Design in Public Environments

In situations in public environments, specifically when each of the various streams of earcon needs to be clearly perceived and understood by individuals, it is important to organize the acoustic features of music based on the psychology of cognitive load. When the timbre of earcon is clearly different acoustically as well as perceptually, the contour directions are not necessarily incongruent. That is, the pitch contour itself is not of a higher value if the distracting music stimuli have different pitch contours and similar timbres. However, when the contours are different, the timbres must be dissimilar to each other. Other than that, there is a very high chance that information from different earcons will be integrated, forming a single percept [45,91].

Timbre refers to the quality that makes one particular musical sound different from another [97,98,99] even when they have the same pitch. Schröter et al. [100] found that two concurrent pitch contours presented with similar timbres are likely to generate an integrated single percept, indicating auditory redundancy gains. In a similar vein, Galvin, Fu, and Oba [101] stated that direction identification is significantly affected by the level of competition in terms of timbre—the existence of masker sound lowers identification performance, and the performance is even worse when the timbre of the masker becomes stronger. This implies that earcons are perceived and understood without any additional increases in cognitive load and thus enhance effective and efficient decision-making and action when two concurrently present earcons have congruent/incongruent directional information and dissimilar timbres.

This study has several limitations, including the small sample size (*N* = 16) and the skewed female-to-male ratio. Some previous fNIRS studies have reported sex-related differences [102,103,104]. This issue remains controversial, with different findings depending on the areas of behavioral function. For example, the difference is more obvious when emotional aspects are the focus of the study, including emotional stress [104] and cooperation [102]. In this study, we found no sex-related differences in any of the behavioral or hemodynamic responses. However, in future studies, we will directly address this issue by controlling the sample size, sex ratio, and age (note that our study only included participants who were in their early to mid-20s). Additionally, in the present study, we selected synthesized instrument timbres from default MIDI instruments based on an approach of music psychology. Given that timbre is a multidimensional attribute that deals with spectral and temporal features [105], brightness and sharpness—which correspond to the harmonic spectral centroid and envelope attack time—for example, should have been considered in stimulus and task design. In future studies, the multidimensionality of timbre needs to be carefully designed, and the effect of such features on cognitive load changes needs to be investigated.

Another limitation of this study is that neurophysiological responses were measured using fNIRS. Although fNIRS is portable and relatively tolerant to motion, unlike other brain imaging methods [106,107], it measures hemodynamic responses only at the cortical level, while techniques such as functional magnetic resonance imaging and magnetoencephalography, which can measure responses at the sub-cortical level or determine the influence of emotion (e.g., panic and fear), are important in fire evacuation scenarios. Further, fNIRS only examines the frontopolar activation; therefore, the relationships between the PFC and other brain regions have not been included in the current study. Thus, we must be cautious before generalizing that the cognitive thresholds observed in CIT4 and CIT5 are only dependent on the role of the PFC.

## 5. Conclusions

Providing timely, efficient information is critical, and auditory signals are predominantly used to achieve this because they allow simultaneous processing in some contexts. The present study employed a reductionist approach in relation to the basic processing of complex auditory signals. We examined cognitive load changes associated with different types of CITs, which can simulate various types of attention required in a real-world auditory environment. Our findings show that melodic CITs can impose different levels of cognitive loads. Moreover, melodic CITs could be a potentially scalable marker to measure cognitive threshold in terms of auditory perception. The cognitive threshold for young adults can shift, and attention can be divided. Further, additional analysis (i.e., analysis of contour congruence and timbre similarity) revealed that appropriate tuning of the relationship between timbre and pitch contour can lower the perceived cognitive load and, thus, can be an effective design strategy for earcon in a public environment, even with concurrent streams without cognitive overload. As a concluding remark, we hope that, by applying the music-based tasks proposed in this article to other conditions and contexts, researchers can provide the insights required to understand the relationship between music and cognition and, thus, to design a more effective and esthetic nonverbal auditory earcon that is applicable to public auditory environments.

## Figures and Tables

**Table 1:** Structure of the contour identification tasks (CITs).

CIT	Target	Distractor	Given Task	Cognitive Load
1	Melodic contour	None	Focus	Low 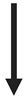 High
2	Melodic contour	Environmental sounds	Focus	
3	Melodic contour	Target-like contours	Select	
4	Melodic contour	Target-like contours	Shift	
5	Melodic contour	Target-like contours	Divide	

**Table 2:** Behavioral responses across the CITs (*N* = 16).

CITs	Characteristics	Accuracy		Reaction Time (ms)	
		Mean	SD	Mean	SD
1	Focused identification task	0.97	0.11	2895	842
2	Focused identification task against noise	0.96	0.11	2489	785
3	Selective identification task	0.92	0.11	2998	1443
4	Alternating identification task	0.89	0.12	3906	902
5	Divided identification task	0.67	0.17	7825	2292

CIT: contour identification task; SD: standard deviation.

**Table 3:** Descriptive statistics across CITs.

CIT	Oxy/Deoxygenation	CH7		CH8		CH9		CH10	
		M	SD	M	SD	M	SD	M	SD
1	HbO_2_	0.036	0.028	0.002	0.024	0.032	0.020	0.002	0.030
	HHb	−0.066	0.019	−0.086	0.025	−0.056	0.022	−0.076	0.025
2	HbO_2_	0.034	0.027	0.000	0.024	0.032	0.02	0.000	0.029
	HHb	−0.066	0.018	−0.087	0.024	−0.056	0.022	−0.077	0.025
3	HbO_2_	0.033	0.028	0.003	0.022	0.036	0.02	0.003	0.029
	HHb	−0.071	0.018	−0.086	0.024	−0.057	0.023	−0.080	0.023
4	HbO_2_	0.034	0.026	0.013	0.028	0.032	0.018	0.011	0.031
	HHb	−0.075	0.022	−0.090	0.028	−0.062	0.024	−0.080	0.028
5	HbO_2_	0.04	0.03	0.014	0.027	0.051	0.022	0.026	0.038
	HHb	−0.071	0.020	−0.075	0.028	−0.057	0.024	−0.073	0.028

CIT: contour identification task; CH: channel; M: mean; SD: standard deviation; HbO_2_: oxygenated hemoglobin; HHb: deoxygenated hemoglobin.

**Table 4:** Accuracy based on timbre similarity and direction congruence in CIT3 and CIT4.

Timbre Similarity	Direction Congruence	Number of Items	Mean	SD
Similar	Congruent	27	1.00	0.00
	Incongruent	176	0.84	0.37
Dissimilar	Congruent	69	0.97	0.17
	Incongruent	340	0.95	0.21

CIT, contour identification task; SD, standard deviation.
